# Buchbesprechung zu Immediatszeitungsberichten (Stöber et al. 2018 und Hoppe et al. 2020)

**DOI:** 10.1007/s11616-020-00607-2

**Published:** 2020-09-25

**Authors:** Jürgen Wilke

**Affiliations:** Institut für Publizistik, Johannes Gutenberg-Universität Mainz, Jakob Welder-Weg 12, 55126 Mainz, Deutschland

## Sammelrezension

1. *Hoppe, Albrecht/Neitmann, Klaus/Stöber, Rudolf (Hrsg.)*: Die Immediatzeitungsberichte der Potsdamer Regierungspräsidenten 1867–1914. Eine Kommentierte Edition in vier Bänden. Bremen: edition lumière 2020. CXX + 3566 Seiten. Preis: € 44,80 je Band
2. *Stöber, Rudolf/Umscheid, Florian Paul*: Politische Interessenkommunikation in der Modernisierung. Das Beispiel des Regierungsbezirks Potsdam (1867–1914). Bremen: edition lumière 2018. 475 Seiten. Preis: € 44,80

Bei den hier anzuzeigenden fünf Bänden handelt es sich in jeder Hinsicht um ein *opus magnum*. Sie bringen zusammen 7,6 Kilo auf die Waage, nehmen gut ein Viertel Regalmeter in Anspruch und summieren sich auf mehr als 4000 arabisch nummerierte Seiten plus weitere mehr als hundert in altrömischer Zählung. Der erste, bereits 2018 erschienene wird von den Autoren als „Begleitband“ deklariert, bei den vier anderen handelt es sich jetzt um die dazugehörige Quellenedition. Dass es sich um ein außerordentliches Werk handelt, dafür spricht auch seine Entstehungsgeschichte. Sie erstreckt sich über ein Jahrzehnt, inklusive eines mehrjährigen DFG-Projekts. Überdies handelt es sich um das zweite von Rudolf Stöber initiierte Unternehmen zur Erschließung zentraler Quellen der amtlichen preußischen Kommunikationspolitik der zweiten Hälfte des 19. Jahrhunderts. Bereits zuvor hat er eine Digitalisierung zweier amtlicher Steuerungsinstrumente, der „Provinzial-Correspondenz“ (1863–1884) und der ihr nachfolgenden „Neuesten Mittheilungen“ (1882–1894), vorangetrieben. Und auch dies ist nur Teil des schon in seiner Habilitationsschrift („Die erfolgverführte Nation“, 1998) zutage getretenen Interesses an den historischen Mitteln der Beobachtung von Öffentlichkeit.

Im jetzigen Fall geht es um die so genannten Immediatzeitungsberichte. Das waren keine gedruckten Zeitungen, wie man leicht missverstehen könnte, sondern geschriebene Mitteilungen, welche die Präsidenten in den Landkreisen Preußens vierteljährlich direkt („immediat“) für den König und die obere Ministerialbürokratie abzuliefern hatten. Wenn dergleichen „Zeitungen“ genannt wurde, so im altertümlichen Sinne dieses Begriffes als Nachricht oder Neuigkeit. Solche Berichte gab es in Preußen schon seit dem 18. Jahrhundert. Doch ihre Bedeutung wuchs im 19. Jahrhundert, vor allem als 1867 für sie neue amtliche Regeln eingeführt wurden. Von da an verfolgen Stöber und Umscheid die Entwicklung der IZB bis zu ihrer Einstellung im ersten Weltkrieg. Und zwar tun sie das exemplarisch für den Regierungsbezirk Potsdam, den sie wegen seiner herausragenden und für den sozialen Wandel symptomatischen Bedeutung in der gesellschaftlichen Modernisierung ausgewählt haben.

In geradezu mustergültiger Weise werden in der Studie mehrere für moderne kommunikations- und medienhistorische Untersuchungen essentielle Dinge in der Studie zusammengeführt: Eine kommunikations- und medientheoretisch inspirierte Fragestellung, die an den Begriff „Interessenkommunikation“ und die zentrale Rolle der Bezirksregierung als Interpret, Übersetzer und Mediator der politischen Willensbildung anknüpft. Damit erfüllten die IZB eine zentrale Funktion im politisch-administrativen Apparat. Zweitens eine historisch-quellenkritische Evaluierung des untersuchten Materials. Drittens eine Methodenkombination, nämlich die Verbindung von qualitativ-hermeneutischen und empirisch-quantitativen Verfahren. Und dies alles wird angewandt auf ein bisher allenfalls am Rande wahrgenommenes Mittel der politischen Kommunikation, das damit überhaupt erst richtig ins historische Bewusstsein gehoben wird. Während die Quellenkritik problembewusst zu klären versucht, wie man die Immediatzeitungsberichte nach Intentionen, Entstehung und Inhalten eigentlich zu interpretieren hat, stützen sich die Autoren für die systematische Inhaltsanalyse auf mehrere Codebücher und einen Begriffsthesaurus. Die Ergebnisse der Erhebungen werden entsprechend in einer Vielzahl von Schaubildern und Tabellen verdichtet.

Nach der Explikation der Fragestellung und dem gewählten Untersuchungsrahmen folgt zunächst ein Kapitel zu den Grundbedingungen der politischen Kommunikation in Preußen (Pressewesen, Vereinslandschaft, Parteien). Danach wird geschildert, wie die IZB, die von den Regierungspräsidenten anzufertigen waren, zustande kamen. Sie basierten auf Zulieferungen der unteren Verwaltungsbehörden, insbesondere der Landräte. Dieses Abhängigkeitsverhältnis ist ein eigener Gegenstand der Untersuchung und der Quellenedition. Das vierte Kapitel bietet einen Überblick über die Struktur und die Themen der Immediatzeitungsberichte, bevor die Hauptthemen im Einzelnen abgehandelt werden. Nach den amtlichen Vorgaben erwartete man von den Berichten Informationen zu den Bereichen Landeskultur, öffentliche Bauten, öffentliche Stimmung und Militärverhältnisse sowie inoffiziell zu Industrie, Handel und Gewerbe. Neben diesen Kernrubriken kamen jedoch unregelmäßig noch andere Felder des gesellschaftlichen Lebens vor (z. B. Gesundheitszustand, Unfälle und Verbrechen). Insgesamt waren es 18. Es fällt schwer, aus der Vielzahl der Befunde der Themenanalyse hier einzelne herauszugreifen. Viele sind interessant und vertiefen unsere historischen Kenntnisse. Den Kommunikationswissenschaftler dürfte insbesondere interessieren, was die Berichte zu den Wahlen zum Reichstag und zum Preußischen Abgeordnetenhaus mitteilten. Die Verfasser registrieren hier geradezu eine „Obsession gegen die Sozialdemokratie“. Den heute in der Wahlkampfberichterstattung häufig beobachteten Negativismus gab es offenbar nicht. Eine Dominanz positiver Selbstdarstellung, wie man vielleicht erwarten könnte, boten die IZB aber auch nicht. Jedenfalls darf man bei der Lektüre nicht vergessen, dass es sich hier nicht um Produkte des politischen Journalismus, sondern um amtliche Funktionstexte handelte. Instruktiv sind auch die Aussagen zur „Öffentlichen Stimmung“, auch sie eine „Königsrubrik“, und dies gerade im Zeitverlauf. Dieser Teil der Darstellung enthält einen Rückbezug auf Stöbers frühere einschlägige Untersuchung, ein seltener Fall, wo ein Autor eine Art Selbstprüfung unternimmt. Neben diesen Befunden stehen viele eher sozialgeschichtlich relevante, beispielsweise zu Streiks und Arbeitskämpfen. Um einen aktuell bemerkenswerten hervorzuheben: Bei neuen Bau- und Infrastrukturprojekten gab es seinerzeit keinen öffentlichen Widerstand. Wutbürger traten damals nicht auf. Der Modernisierung begegnete die Bevölkerung – nach amtlicher Beobachtung – aufgeschlossen.

In der Quellenedition sind in den ersten drei Bänden die 186 zwischen dem 29. Januar 1868 und dem 7. Mai 1914 datierten Immediatzeitungsberichte der Potsdamer Regierungspräsidenten abgedruckt. Das wurde in Kooperation mit dem Brandenburgischen Landeshauptarchiv realisiert, dessen Leiter Klaus Neitmann die Publikation mit verantwortet. Deren eigentlicher Gewinn besteht aber darin, dass diese Berichte außer mit einer längeren synthetisierenden Einleitung mit einem ausgiebigen Kommentarteil versehen sind, der von Albrecht Hoppe erarbeitet wurde. Nicht genug zu würdigen ist, was er mit enzyklopädischer Perfektion an Hintergrundinformationen zum Verständnis der IZB zusammengetragen hat. Diese verteilen sich auf rund 1500 teilweise ausführliche Fußnoten. Kaum eine substanzielle Information entbehrt der Fundierung. Und einbezogen in die Kommentierung werden auch, so weit vorhanden, die Vorlagen, aus denen die Regierungspräsidenten bei der Verfertigung der IZB schöpfen konnten. Erst wenn man diese mit speziellem Blickwinkel durchforstet, wird man den Wert der Kommentierung voll abschätzen können.

Der Wert einer solchen Quellenedition erweist sich erst durch ihre Erschließung. Dafür ist der vierte Band der Quellenedition maßgebend und unentbehrlich, der ausschließlich Nachweise und Register enthält. Er allein hat einen Umfang von 690 Seiten und verzeichnet neben den Archivalien und der Literatur, die herangezogen wurden, eine ganze Reihe von Registern. Die IZB wurden nicht nur nach Orten, Ländern, Personen, Firmen und Gesetzen aufgeschlüsselt. Eine Detailsuche ermöglichen vor allem ein Sachregister und ein Stichwortverzeichnis. Während das letztere auf der Ebene der Einzelbegriffe verbleibt, ist das erstere alphabetisch nach thematischen Sachgruppen gegliedert, die jeweils mehr oder weniger Spezifizierungen aufweisen.

Kaum jemand wird die Quellenedition fortlaufend von vorne bis hinten lesen wollen. Man wird sie gleichwohl auf verschiedene Weise nutzen können. Mit ihrer Hilfe kann man die im Begleitband gegebene Darstellung und Analyse überprüfen und erweitern. Vielleicht schlägt man aber auch einfach einmal stichprobenweise nach, was an diesem oder jenem Tag im Untersuchungszeitraum im Regierungsbezirk Potsdam so alles los war. Oder man folgt den Einträgen im Schlagwortverzeichnis und sucht gezielt nach dem Vorkommen bestimmter Themen. Beispielsweise unter Wahlen oder unter Presse/Zeitung/Journalismus, wozu auch schon Stöber und Umscheid die Quelle ausgewertet haben. Oder in Zeiten des im Jahre 2020 aktuell grassierenden Corona-Virus könnte man unter Krankheiten/Epidemien/Seuchen nachschlagen und würde einiges über Diphterie, Masern, Scharlach und Typhus als die seinerzeit vorherrschenden Infektionen (und den Umgang mit ihnen) erfahren.

Eine Frage, die letztlich offenbleibt, ist freilich, was die Adressaten der Berichte, primär die preußischen Könige, mit den Immediatzeitungsberichten anfingen. Dies interessiert umso mehr, als deren Anfertigung sehr aufwändig war und sie deshalb auch schließlich eingestellt wurden. Mündete ihre Kenntnisnahme in politisches oder administratives Handeln? Nicht nur dies könnte, wenn denn rekonstruierbar, Gegenstand einer weiteren (zumindest exemplarischen) Untersuchung sein. Denkbar wäre auch, die IBZ als äußere Folie für eine Studie zur gleichzeitigen Presseberichterstattung zu nehmen, wobei amtliche und journalistische Realitätsdarstellung und die ihnen zugrunde liegenden Selektionskriterien im Kontrast deutlich werden könnten.

Wahrscheinlich werden Werke wie das hier angezeigte in Zukunft gar nicht mehr in gedruckter Form erscheinen. Dazu gehört heute schon ein noch der Gutenberg-Galaxis verpflichteter Verlag wie die Bremer edition lumière. Im Zeitalter der fortschreitenden Digitalisierung aber wird dergleichen in absehbarer Zeit nur noch am Bildschirm zu konsumieren sein. Wer das in seiner drohenden Ausschließlichkeit nicht liebt, der kann an diesen Bänden noch seine Freude haben.

